# On the peculiar morphology and development of the hypoglossal, glossopharyngeal and vagus nerves and hypobranchial muscles in the hagfish

**DOI:** 10.1186/s40851-014-0005-9

**Published:** 2015-01-29

**Authors:** Yasuhiro Oisi, Satoko Fujimoto, Kinya G Ota, Shigeru Kuratani

**Affiliations:** Laboratory for Evolutionary Morphology, RIKEN, 2-2-3 Minatojima-minami, Chuo-ku, Kobe, Hyogo 650-0047 Japan; Laboratory of Aquatic Zoology, Marine Research Station, Institute of Cellular and Organismic Biology, Academia Sinica, No. 23-10, Dawen Road, Jiaoxi, Yilan 26242 Taiwan

**Keywords:** Agnathans, Cranial nerves, Hypobranchial muscles, Cyclostomes, Embryo, Evolution, Hagfish

## Abstract

**Introduction:**

The vertebrate body is characterized by its dual segmental organization: pharyngeal arches in the head and somites in the trunk. Muscular and nervous system morphologies are also organized following these metameric patterns, with distinct differences between head and trunk; branchiomeric nerves innervating pharyngeal arches are superficial to spinal nerves innervating somite derivatives. Hypobranchial muscles originate from rostral somites and occupy the “neck” at the head-trunk interface. Hypobranchial muscles, unlike ventral trunk muscles in the lateral body wall, develop from myocytes that migrate ventrally to occupy a space that is ventrolateral to the pharynx and unassociated with coelomic cavities. Occipitospinal nerves innervating these muscles also extend ventrally, thereby crossing the vagus nerve laterally.

**Results:**

In hagfishes, the basic morphological pattern of vertebrates is obliterated by the extreme caudal shift of the posterior part of the pharynx. The vagus nerve is found unusually medially, and occipitospinal nerves remain unfasciculated, appearing as metameric spinal nerves as in the posterior trunk region. Moreover, the hagfish exhibits an undifferentiated body plan, with the hypobranchial muscles not well dissociated from the abaxial muscles in the trunk. Comparative embryological observation showed that this hagfish-specific morphology is established by secondary modification of the common vertebrate embryonic pattern, and the hypobranchial muscle homologue can be found in the rostral part of the oblique muscle with pars decussata.

**Conclusion:**

The morphological pattern of the hagfish represents an extreme case of heterotopy that led to the formation of the typical hypoglossal nerve, and can be regarded as an autapomorphic trait of the hagfish lineage.

## Introduction

Although the extant jawless vertebrates—the hagfishes and lampreys—represent two groups belonging to a monophyletic taxon, Cyclostomata [1-9], and sharing a common pattern of embryogenesis [10], their late embryonic and adult stages exhibit very different anatomical patterns, as seen by the position of the postotic pharyngeal arches, the morphology of the oral apparatus, and the presence or absence of a nasopharyngeal duct [10,11]. Most of the morphological differences between these two groups stem from a derived pattern of development occurring in the late phase of hagfish ontogeny, making this animal group appear highly exceptional among all vertebrates [10].

The autapomorphic traits in the hagfish are generally reasonably explained as morphological shifts or modifications of embryonic structures commonly found in the hagfish and the lamprey [10]. For example, the nasopharyngeal duct unique to the hagfish arises initially as a blind sac similar to the nasohypophyseal duct in the lamprey. This duct in the hagfish, however, secondarily grows caudally, and as a result of the degeneration of the root of the post-hypophyseal process, or the homologue of the ammocoete upper lip, the blind sac becomes confluent with the pharynx. The posterior shift of the pharynx in the hagfish can also be ascribed to the growth of that part of the pharynx corresponding to the third pharyngeal arch [10,12,13]. Importantly, even after the dynamic changes introduced into the late embryogenesis of the hagfish, the relative topographical relationships between organs and anatomical elements, once established at pharyngular stages as the pan-cyclostome embryonic pattern, do not usually change [10]. Thus, by revisiting the pan-cyclostome pattern as a reference, morphological homologies can be well established between the lamprey and hagfish, no matter how far the hagfish body may have deviated from that of the cyclostome ancestor [11].

**Figure 1:**
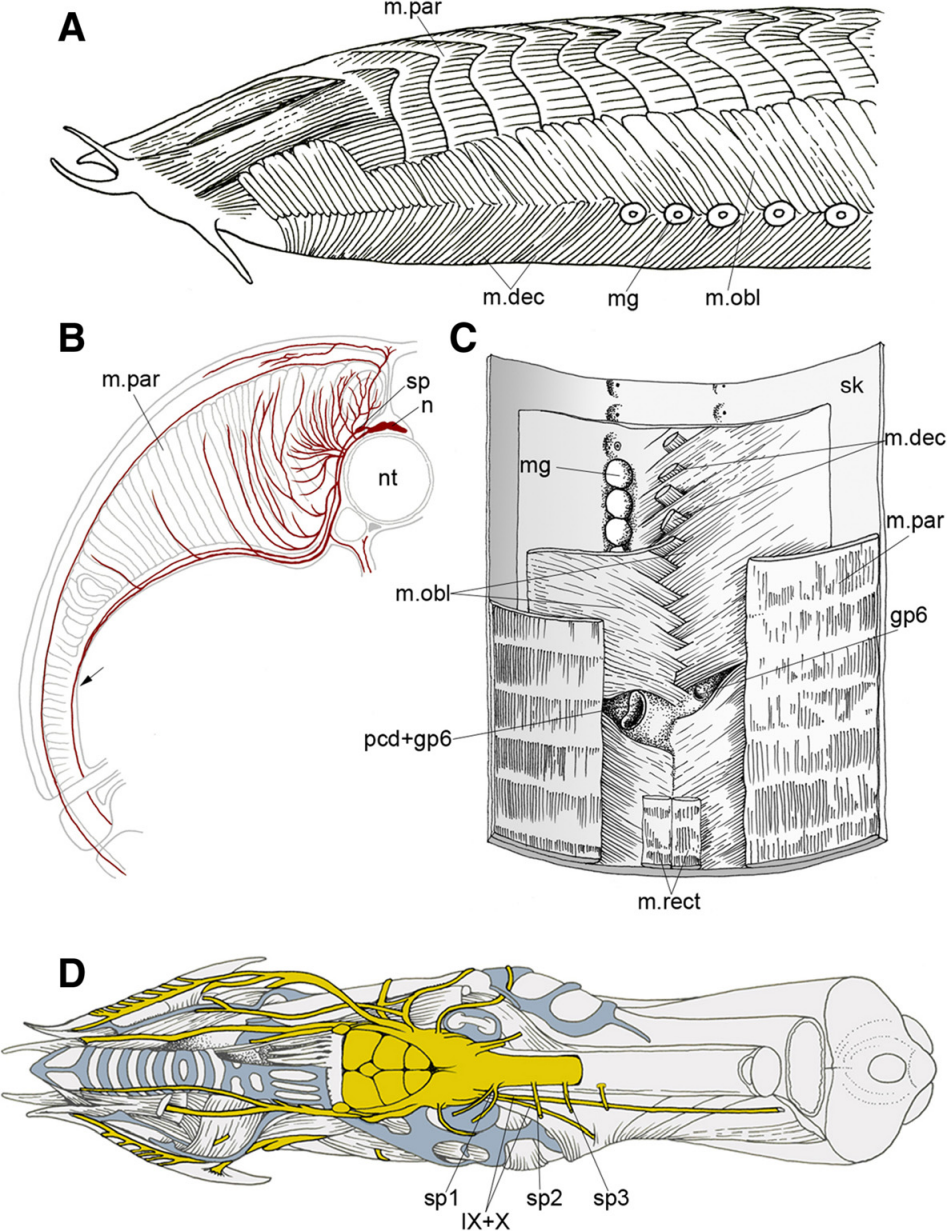
**Anatomical configuration of the putative “neck” region of the hagfish. (A)** Left lateral view of an adult hagfish, *Myxine garmani*, originally drawn by Nishi (1938). **(B)** A schematized illustration showing the innervating pattern of a spinal nerve in a transverse section of a hagfish, *Bdellostoma dombeyi*. Redrawn from [22]. The arrow indicates the ventral branch that extends ventrally to innervate the oblique and rectus muscles. **(C)** Dorsal view of the oblique and rectus muscles together with the skin (sk) in *M. glutinosa*. **(D)** Dorsal view of a dissected head of *M. glutinosa* showing the morphological patterns of the nervous system (yellow). Note that the spinal nerves (sp1–3) are located dorsolateral to the branchiomeric nerve components: the glossopharyngeal (IX) and vagus (X) nerves. Light blue indicates cranial cartilage. **(C)** and **(D)** are redrawn from [15].

Other hagfish-specific unique morphologies are encountered in the topography of some cranial nerves and muscles at the anatomical level (Figure 1). Like lampreys, hagfishes seem to possess homologues of hypobranchial muscles in the ventrolateral aspect of the pharyngeal wall, although these are not well defined [14-16]. However, the occipitospinal nerves innervating these muscles in the hagfish do not form a bundle as would a typical hypoglossal nerve that passes caudally to circumvent the pharynx, or along the circumpharyngeal contour representing the head-trunk interface [17]. Instead, they grow ventrally as individual segmental nerves to reach the hypobranchial muscles (Figure 1D; see below). In jawed vertebrates, spinal nerves never grow into the pharyngeal arch domains; for instance, the hypoglossal nerve passes between the fork formed by the accessory and vagus nerves, terminating in a more superficial position than the vagus [18].

This topographical inconsistency in peripheral nerve morphology between the hagfish and other vertebrates apparently violates the conserved topographical relationships that assure morphological homology, and is likely an example of the evolutionary novelties that are often associated with topographical mismatch [19,20]. In the same manner as the shifted position of the turtle’s scapula, which is encapsulated in the ribcage in association with the acquisition of the carapace, the peculiar positions and morphologies of the occipitospinal nerves in the hagfish are likely associated with the unusually caudal position of the pharynx of this animal. Given the pan-cyclostome embryonic pattern in the organogenetic period of cyclostomes, it is difficult to imagine how this hagfish-specific pattern could have arisen during development. This is especially so given that the morphological patterns of the developing muscles and cranial nerves are primarily identical, even between the lamprey and jawed vertebrates [10,11]. Thus, to explain the peculiar morphological pattern, it seems necessary to assume that a certain fundamental shift was introduced specifically in the hagfish developmental program.

The anatomical configuration of the muscles and nerves in the neck-like region of the adult hagfish (the domain between the cranium and the posteriorly shifted pharynx) has been described by Müller [21,22], Nishi [23], and Marinelli and Strenger [15] (Figure 1), and was confirmed in the dissection of adult *E. burgeri* in the present study. The following summary is mainly a review of previous studies, presented here to elucidate the anatomical pattern of the hagfish muscular system.

Along almost the entire body axis, the lateral aspect of the hagfish body exhibits dorsal segmental muscles, or myotomes, known as the musculus (m.) parietalis [15,23], and ventral, thinner plates of muscles named m. obliquus that show no overt segmental pattern [24] (Figure 1A, C). The m. obliquus extends from just caudal to the mouth to the cloaca. More superficial muscle covers the rostral and ventral part of the oblique muscle, extending caudally to the level of the pharyngeocutaneous duct or the caudalmost gill pore (dissection of *E. burgeri* in the current study showed six gill pores on both sides, with the caudalmost gill pore on the left side being confluent with the pharyngeocutaneous duct). This superficial muscle represents the ventral ends of the oblique muscle fibers that originate from the contralateral side. Thus, ventral ends of left and right oblique muscle fibers interdigitate along the ventral midline, extending to the contralateral side. These contralateral myofibers end roughly at the level just below the mucous glands (Figure 1A, C). Thus, the oblique muscle becomes two-layered ventral to the mucous pores, each layer distinguishable by the direction of its muscle fibers [14,23]. Of these, the ventral moiety was specifically called “pars decussata” by Nishi [23]; it appears to be an outer, different layer of muscle separated from the ipsilateral oblique muscle, present only in the rostral part of the body with its caudal end found close to the last gill pore [23]. Thus, the oblique muscle appears two-layered only in the pharyngeal region and more anteriorly (Figure 1A).

In transverse section, the parietal muscle appears as a muscle plate, as seen in aquatic vertebrates, especially resembling that of the lamprey, whereas the oblique muscle lies in a more superficial position, with its dorsal portion covering the ventral part of the parietal muscle. The anatomical configuration of these muscles and their topographical relationships have been described by Marinelli and Strenger [24] and Nishi [23] (Figure 1). Attached to the inner aspect of the m. obliquus, and medial to the series of mucous glands, there is another pair of muscles, m. rectus, running longitudinally close to the ventral midline (Figure 1C). For most of its axial length, this muscle lies dorsal to the oblique muscle plates, but rostrally it becomes thicker laterally and detached from the obliquus, with its termination at a cartilage of the lingual apparatus, cartilago linguae basalis pars media [11,23,24]. Caudally, the rectus muscle merges into the cloacal sphincter [23]. Unlike the oblique muscles, whose fibers run more or less transversely, fibers of rectus muscle run longitudinally, and the entire muscle is apparently segmented as the parietal muscle plate (Figure 1C). In terms of relative positions, innervation patterns (see below) and connections, the rostral parts of the oblique and rectus muscles in the hagfish resemble hypobranchial muscles in other vertebrates (for homology, see Discussion). The rostralmost part of the hagfish obliquus in particular conspicuously resembles the hypobranchial muscle of the lamprey [25-28] (see below).

All of the above muscles, including the parietal muscle, are innervated by segmental spinal nerves that run ventrally along the medial aspect of these muscle plates, as described by [24] (Figure 1B). The parietal muscle is supplied by small branches of the spinal nerves, issuing from the dorsal and proximal parts of the nerve trunk [23] (Figure 1B). In contrast, the rectus is innervated by the most distal branch of the nerve that bifurcates approximately at the level corresponding to the ventral edge of the parietal muscle, from the branch other than that innervating the oblique muscle [23].

Such close relationships between the nerve branches described above again imply similar morphological properties and developmental origins of the muscles innervated by these nerves, standing in contrast to the parietal muscle. Curiously, there are no spinal nerves that decussate the vagus nerve caudal to the caudalmost pharyngeal pouch to reach the ventral muscles, as found in the occipitospinal (or hypoglossal) nerves in other vertebrates (including the lamprey). Rather, these spinal nerves run superficial to the position of the vagus and glossopharyngeal nerves that pass unusually medially as compared to those in other vertebrates (Figure 1D). For the entire length of its course, the vagus nerve, as well as the glossopharyngeal nerve, is found medial to the somatic muscle plates together with the spinal nerve, a topographical relationship that is opposite to the morphological patterns of these nerves in the neck region of “normal” vertebrates [17] (Figure 1D).

The objective of the present study was to observe the late embryonic period of the hagfish species *Eptatretus burgeri* and *E. atami*, specifically focusing on the development of the caudal pharynx, vagus nerve, and the hypobranchial muscles and associated innervating rostral spinal nerves, to better understand the nature of hagfish-specific developmental repatterning. We found that the developmental anlage of the hypobranchial muscles and the nerve branches innervating them is relatively late in the hagfish, allowing the nerves to take “short-cuts” to innervate the muscles, resulting in the unique anatomical pattern in the hagfish. This phenomenon can be seen as a heterochronic retardation of the hypobranchial system that triggers a heterotopic shift of structures, resulting in the violation of vertebrate anatomical rules. We also recognize and discuss the similarity between the caudal shift of the caudal pharynx in the hagfish and the elongation of the amniote neck from comparative embryological viewpoints.

## Materials and methods

### Sample collection and histological preparation

*Eptatretus burgeri* embryos were collected and fixed as described previously [11,29]. Hagfish embryos were staged according to the method of Dean [30]. For histological sections, we used Kawamoto’s film method and a Paraffin Section Preparation Kit (Section Lab Co. Ltd.; see http://section-lab.jp/English.htm; see also [10,11]). Histological images were recorded with a DP70 digital camera (Olympus Inc., Tokyo, Japan) attached to a light microscope and reconstructed with a computer graphics program (Avizo 3D Visualization Framework, Maxnet, Tokyo, Japan).

### *In situ* hybridization

*In situ* hybridization was performed either by using a standard manual protocol or an automated instrument (Ventana; Roche, Japan). In the standard protocol, serial sections were fixed for 10 min in 4% (w/v) paraformaldehyde in phosphate buffered saline (PBS) at room temperature, washed twice in PBS, treated with proteinase K in 0.01 M Tris buffer (pH 8.0) for 10 min, and then fixed again for 10 min in 4% paraformaldehyde at room temperature. After rinsging twice in PBS, the sections were incubated with 0.25% acetic anhydride and 0.1 M triethanolamine (pH 8.0), washed in PBS, air dried, and hybridized with riboprobes at 51°C for 16–20 h. The sections were then washed in 5× saline sodium citrate (SSC) buffer at 55°C, treated with 50% formamide in 2× SSC at 60°C for 20 min, and then washed once in 2× SSC and twice in 0.2× SSC at 60°C for 20 min for each wash. The sections were blocked with 1.5% blocking reagent (Roche) in 0.1 M Tris buffer with 0.15 M NaCl (pH 7.6), and then incubated with alkaline-phosphatase–conjugated anti-digoxigenin (DIG) antibody (Roche). After final washes of the sections with Tris buffer, positive cells were stained purple with nitroblue tetrazolium salt (NBT) and 5-bromo-4-chloro-3-indolyl phosphate toludinium salt (BCIP). For the automated Ventana instrument, signals were detected and counterstaining was performed by using a BlueMap NBT/BCIP substrate kit (Roche) and a nuclear fast red-equivalent reagent, ISH RED (Roche), as described previously [29].

### Immunohistochemistry and histochemistry

Histological observations were made on hematoxylin and eosin stained sections (thickness, 6–8 μm). To detect axon bundles, anti-acetylated tubulin was applied to sections. Anti-mouse IgG1 was used as the secondary antibody. All histological images were recorded with a DP70 digital camera (Olympus Inc.) attached to a light microscope.

### Molecular cloning

Cloning of *MyHCA* and *HandA* in *E. burgeri* was performed as described elsewhere [31]. The sequence data were submitted to the DDBJ database (AB915326–915327). To identify the orthologous genes of the isolated fragments, comparable sequence data were surveyed using the NCBI protein database and a BLAST search, and multiple sequence alignments were generated using the CLUSTALW multiple alignment program [32].

## Results

### Embryonic shift of the postotic pharynx and somitic derivatives in hagfish embryos

**Figure 2:**
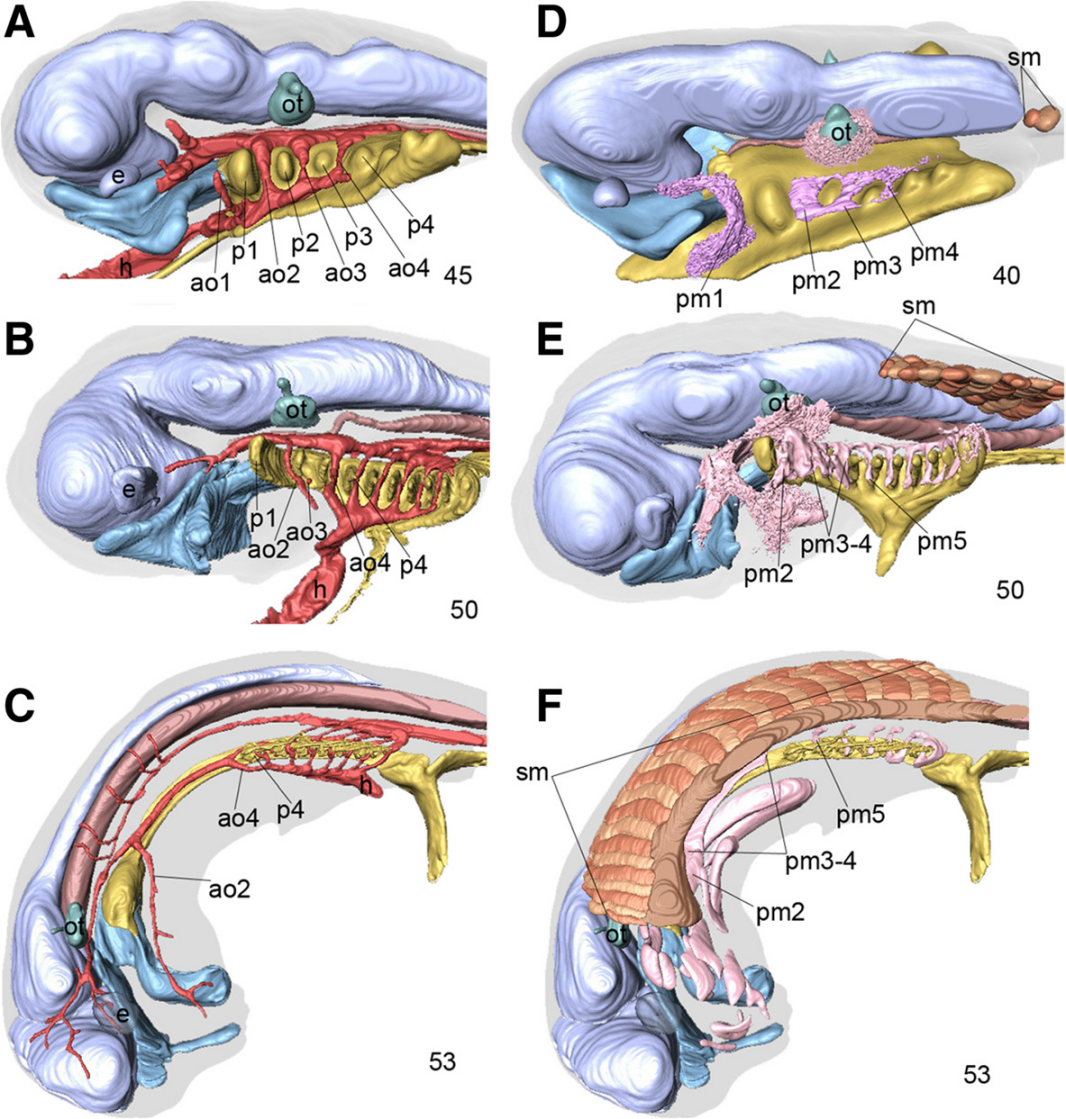
**Mid- to late pharyngular-stage embryos of** ***Eptatretus burgeri*** **. (A–C)** Left lateral views of 3D-reconstructed embryos at stages 45, 50, and 53. Pharyngeal endoderm is colored yellow and the arterial system is colored red. The light blue color beneath the brain primordia represents oronasal ectodermal derivatives (for details, see [10]). Note that the caudal half of the pharynx shifts caudally by the expansion of the pharyngeal arch 3 and 4 domain. **(D–F)** Reconstructions of embryos at stages 40, 50, and 53, with *Tbx1/10A*-positive mesodermal components (colored pink) as well as somites (sm). Note that somites initially arise caudal to the entire pharynx at stage 40, and later shift rostrally to reach the mid-otic level **(F)**.

**Figure 3:**
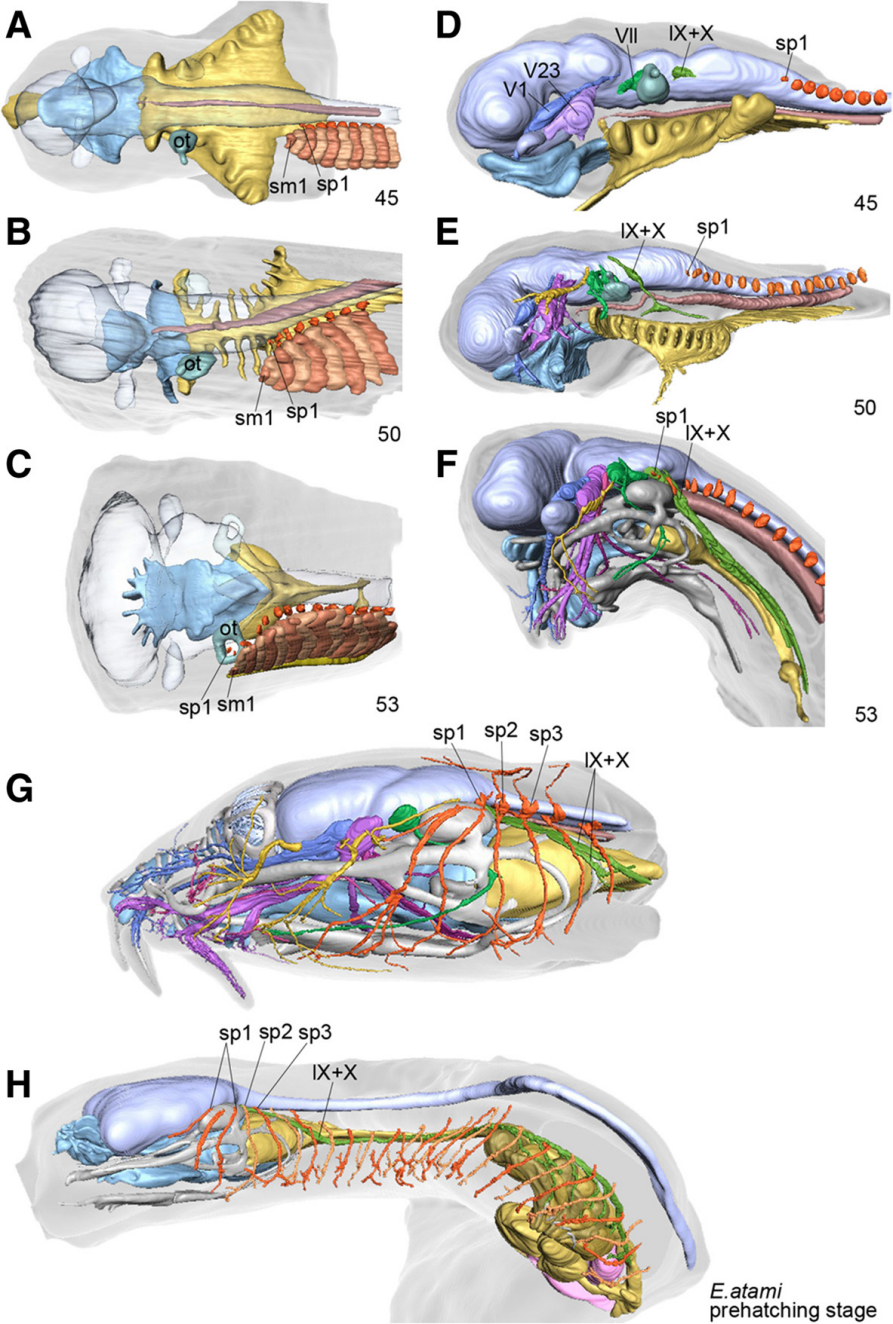
**Mid to late pharyngular-stage embryos of** ***Eptatretus burgeri*** **. (A–C)** Dorsal views of embryos at stages 45, 50, and 53, with the brain and notochord made transparent mainly to visualize the relative positions between pharynx (yellow), otic vesicle (ot) and somites. Dorsal root ganglia of spinal nerves (sp) are shown in orange, medial to the somites. **(D–F)** Development of the peripheral nerves in the same embryo as shown in **A–C**. Cranial nerves are shown by different colors. In this reconstruction, the glossopharyngeal and vagus nerves (green) are not always easy to distinguish from each other. Note that, as with the development of the somites, the spinal nerves first arise caudal to the pharynx and later shift rostrally to the mid-otic level at stage 53. **(G, H)** Reconstructions of the spinal nerves in the head (originally the right side) **(G)** and rostral part the body (head and pharynx; **H**) of a pre-hatching–stage *E. atami* embryo. Spinal nerves are colored orange. Note that in the head the rostral spinal nerves (putative occipitospinal nerves) pass superficial to the branchiomeric nerves, as does the hypoglossal nerve in other vertebrates.

Observations of *E. burgeri* embryos from stages 40 to 53 helped us understand how the hagfish-specific shift of the posterior pharynx takes place (Figures 2 and 3). By stage 50, the anatomical configuration of the hagfish embryo is rather stable (Figures 2A, B, D, E and 3A, B, D, E); at stages 40–45, a total of seven pharyngeal pouches were found on the pharyngeal endoderm, with the otic vesicle appearing dorsal to the second pharyngeal pouch. The rostralmost somite was found caudal to the level of the pharyngeal endoderm (Figure 2D). At stage 50, approximately 10 pharyngeal arches were discernible, all of which were arranged at nearly equal intervals along the anteroposterior axis, indicating no sign of caudal shift of the pharynx by this stage (Figure 2B, E). However, some rostral somites were now found caudal to the fifth pharyngeal arch, and it appeared that not only had the entire pharynx grown caudally, but also that the rostral somites had shifted slightly rostrally (Figures 2E and 3B). These somites correspond to the suprapharyngeal somites in gnathostome embryos [17]. Thus, there are five such somites in a stage 50 embryo of *E. burgeri*, unassociated with a coelomic cavity ventrally.

**Figure 4:**
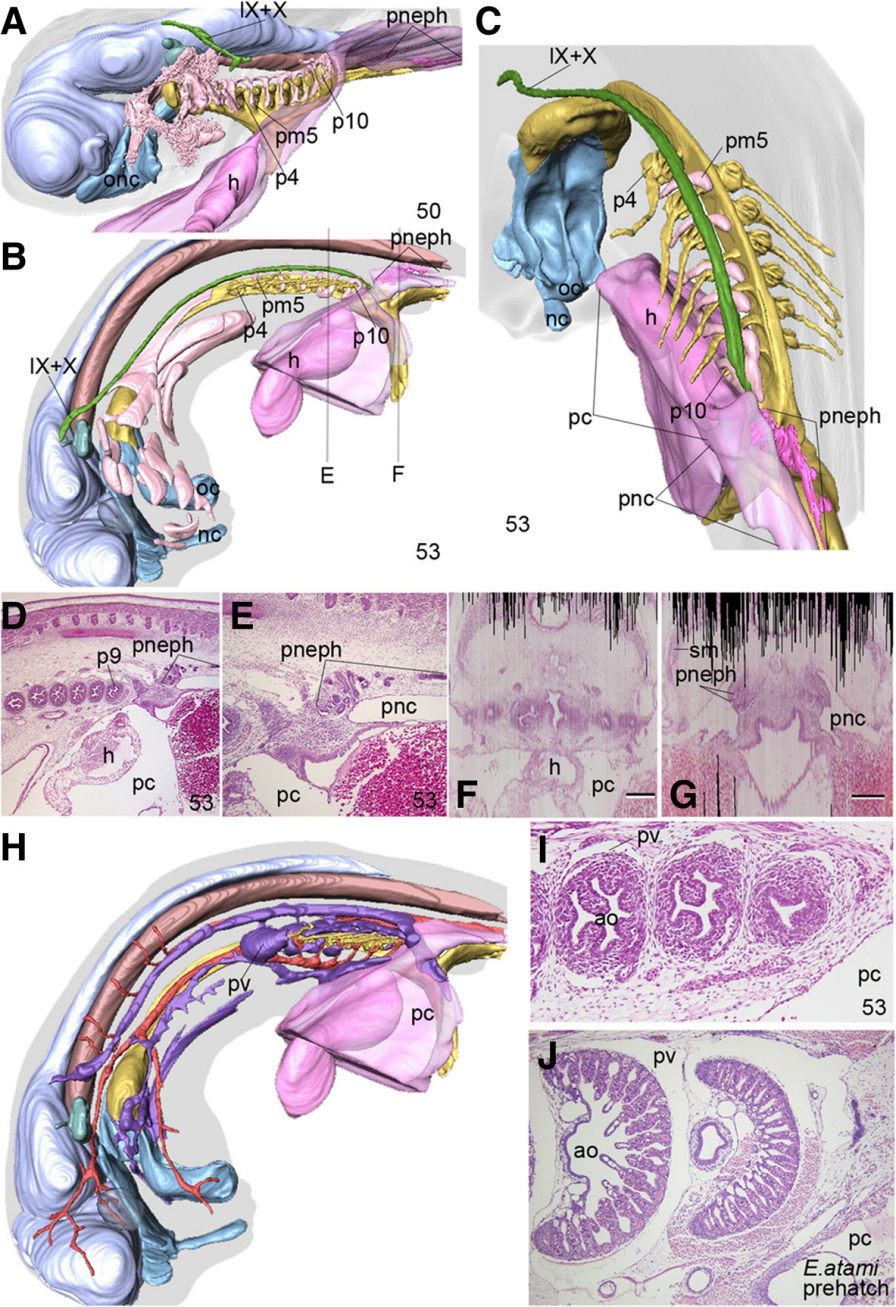
**Developmental changes in the morphology and topography of coelomic cavities. (A)** 3D-reconstructed stage-50 embryo of *Eptatretus burgeri* showing the position of the coelomic cavity (dark pink). Light pink indicates *Tbx1/10A*-positive myoblasts in pharyngeal arches. The coelomic cavity consists of the pericardium and peritoneal cavity, which are well defined and separate from each other at the caudal end of the pharynx. This morphology represents the generalized configuration of the coelomic cavity in vertebrate embryos. **(B, C)** Left lateral **(B)** and left caudal **(C)** views of a stage-53 embryo. Note that the junction between the pericardium (pc) and peritoneal cavity (pnc) corresponds to the level of the 10th pharyngeal pouch (p10) or the caudal end of the posteriorly shifted pharynx. **(D)** Parasagittal section showing the pharyngeal-pericardial region. **(E)** The same section as **C** at higher magnification. Note that the pronephros (pneph) is constantly found in the rostral part of the peritoneal cavity, and caudal to the pharynx. **(F, G)** Two transverse sections cut at the levels shown in **B**, reconstructed from serial parasagittal sections of the stage-53 embryo showing the pericardium (pc in **E**) and peritoneal cavity (pnc in **F**). **(H)** Reconstruction of a stage-53 *E. burgeri* embryo showing the developmental pattern of the venous system (purple). **(I, J)** Parasagittal sections of a stage-53 *E. burgeri* embryo **(I)** and a pre-hatching–stage *E. atami* embryo **(J)** showing the peribranchial venous system. Note that the venous system expands to form a sinus surrounding the gill pouches of the pre-hatching stage, just like a peribranchial coelom.

At stage 50, the coelomic cavities consisted of two distinct portions: the pericardium rostrally and the peritoneal cavity caudally (Figure 4A). The pericardium covered the heart below the pharynx, and continued posteriorly into the peritoneal cavity found caudal to the entire pharynx. Thus the peritoneal cavity was restricted caudal to the pharynx at this stage, and so were the intermediate mesoderm derivatives—the pronephros (Figure 4A, D, E, G).

In hagfish, mesodermal components of the pharyngeal arches do not develop as epithelial coeloms, as found in the shark, but instead form a *Tbx1/10A*-positive mesenchymal core that differentiates into the pharyngeal musculature in late development [10] (Figure 2D–F). The primordium for the peritoneal coelom in the hagfish was also found ventromedial to the dermomyotome, as in gnathostomes (Figure 4G; see below). Such a morphological configuration is common to all gnathostome embryonic patterns, especially resembling that in the pharyngula of the shark, in which the heart primordium appears to be suspended below the pharynx in the organogenetic period [33,34] (reviewed by [17]). However, there was no sign of hypobranchial muscle development by this stage, as normally seen in the hypoglossal cord and formation of the hypoglossal nerve composed of rostral spinal nerves (see below).

**Figure 5:**
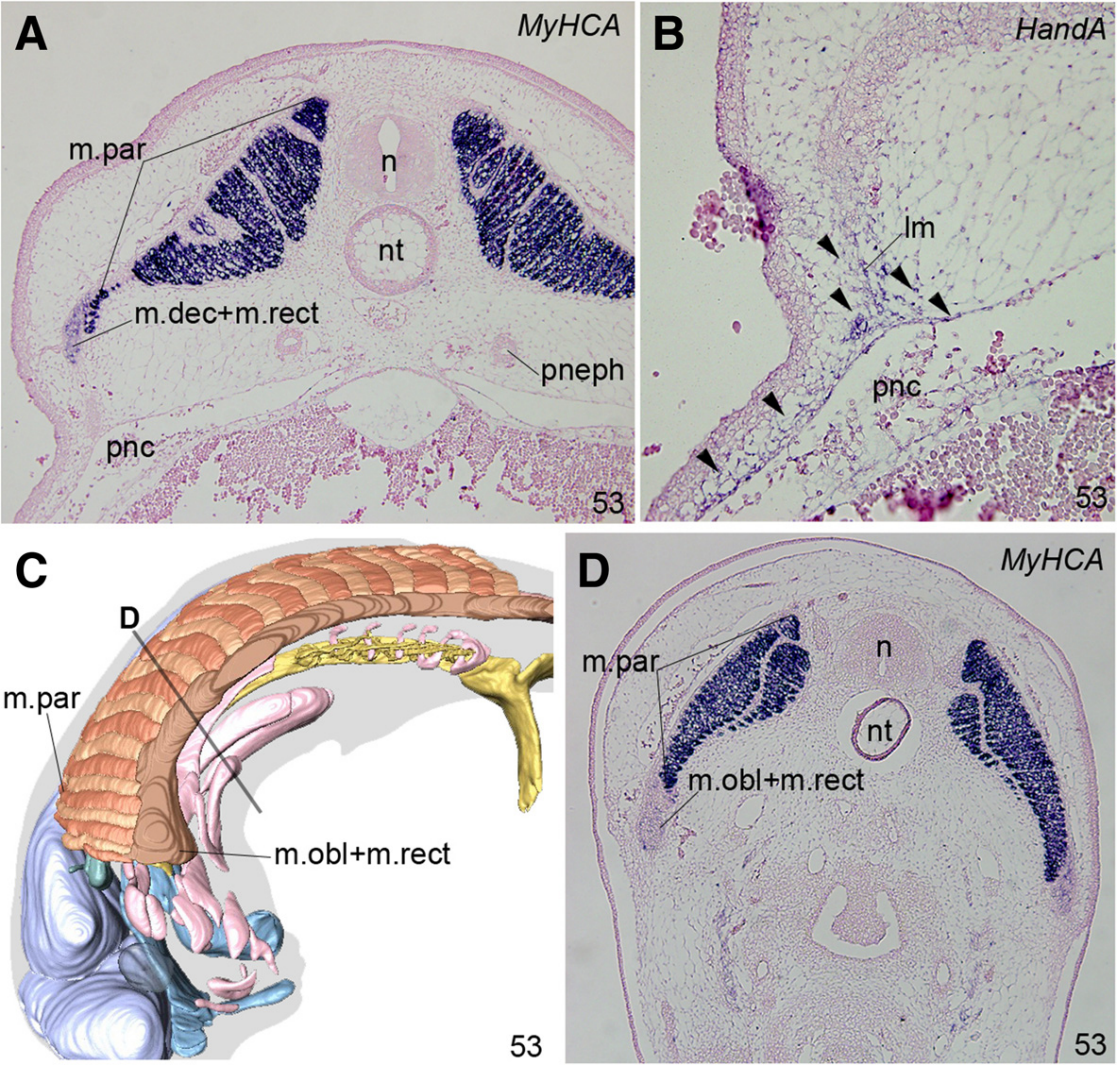
**Histological observations of hagfish muscle development. (A)** Transverse section of a stage-53 *Eptatretus burgeri* embryo, cut at the posterior trunk level. Expression of *MyHCA* was detected by in situ hybridization. This gene was strongly expressed in the myotomal muscle plates (=precursor of the parietal muscles, m.par), and relatively weakly expressed in the abaxial muscles (m.dec + m.rect). **(B)** A transverse section adjacent to **A**, showing the expression of *HandA*, a marker of lateral plate-derived mesenchyme. *HandA*-expressing cells were predominantly located in the lateral body wall (arrowheads). **(C)** Reconstruction of a stage-53 embryo of *E. burgeri* showing the level of the section in **D**. **(D)** A transverse section cut at line D in **C**, showing the relationship between the parietal muscle anlage and the hypobranchial muscle anlage (m.obl + m.rect).

By stage 53, a part of the pharynx corresponding to pharyngeal arch 3 had become extremely elongated along the anteroposterior axis, at the level including the third and fourth pharyngeal arch region (Figures 2C, F; 3C, F; 4B, C, H; 5C; and 6A–C). This is consistent with the fact that this elongated part in the adult contains similarly elongated muscles innervated by the glossopharyngeal nerve [24] (Figure 1D): this nerve consistently innervates the arch 3–derived muscles in jawed vertebrates. This local elongation has been reported to already start by stage 51, when pharyngeal muscle precursors for arches 3 and 4 have expanded anteroposteriorly more conspicuously than those in more posterior arches [10].

**Figure 6:**
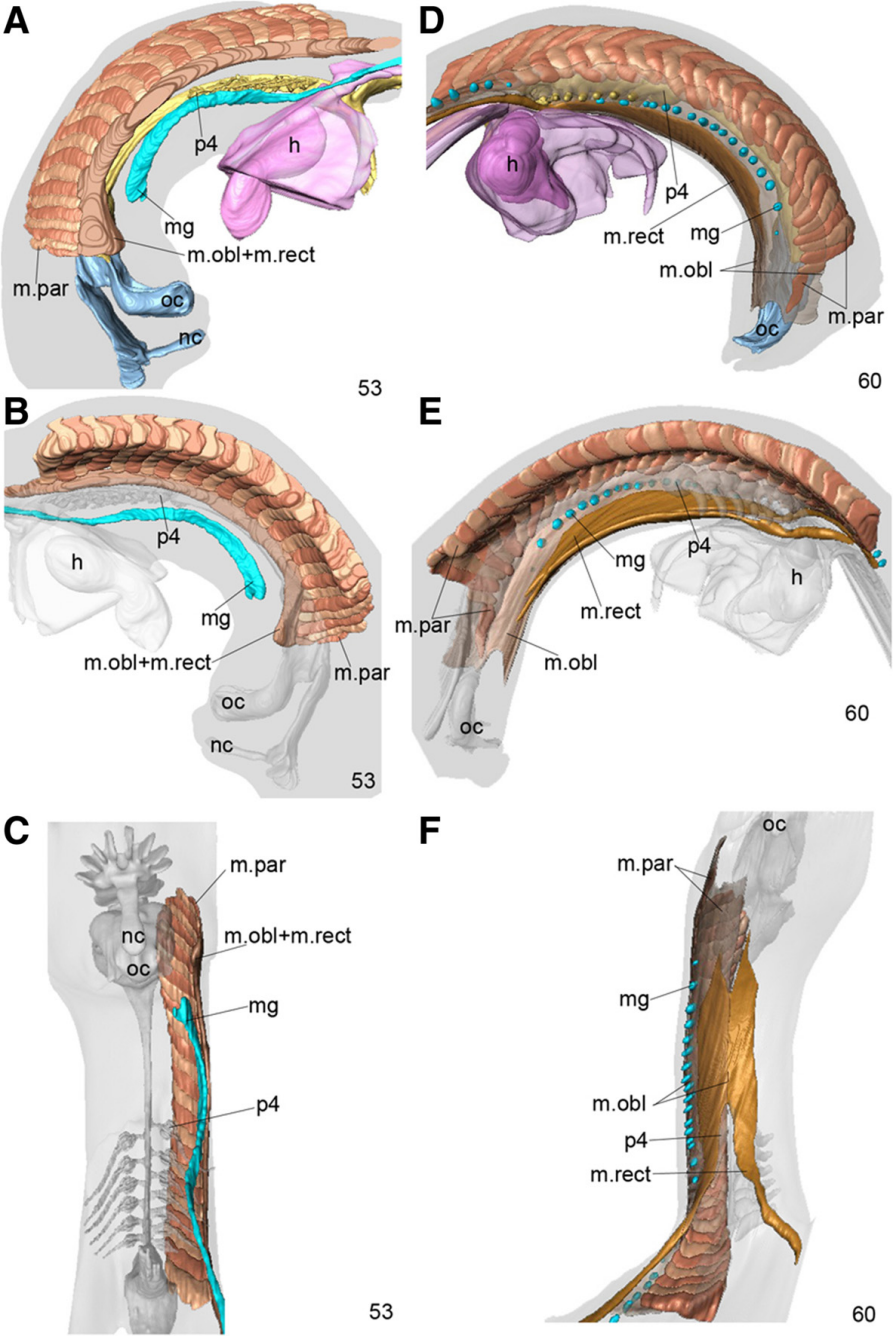
**Development of hypobranchial muscles.** Stage-53 **(A–C)** and stage-60 **(D–F)** embryos of *Eptatretus burgeri* reconstructed to show development of the putative hypobranchial (and abaxial) muscles in the hagfish; left lateral **(A)**, right lateral **(D)**, medial **(B, E)** and ventral **(C, F)** views. At stage 53 **(A-C)**, the rostral part of the common anlage for oblique and rectus muscle (m.obl + m.rect) is arising from the ventrolateral edge of myotomes (anlage of parietal muscles, m.par) to grow ventrally into the superficial layer of the hagfish “neck”, appearing as the basal part of the hypoglossal cord in other vertebrate embryos. The epithelial cord (colored blue) indicates the anlage of the mucous gland (mg). At stage 60 **(D-F)**, the parietal and hypobranchial muscles surround the entire pharyngeal basket laterally **(E)**, and only the gill pores penetrate the muscle to the exterior **(D)**. Mucous glands also develop external ducts that penetrate the hypobranchial muscle in a segmental pattern that does not correlate with that of myotomes **(D)**.

The gill sacs of the posterior pharynx at stage 53 were beginning to be surrounded by venous sinus (Figure 4H, I), which by the pre-hatching stage had become extensively developed (compare Figure 4I to J). This venous sinus appears to specifically facilitate the movement of the gills in the hagfishes.

Concomitant with this elongation, the posterior pharynx as a whole (pharyngeal pouch 4 and posterior ones) had shifted caudally to the mid-trunk region (Figure 2C, F). By this stage, the pharyngeal pouches had become spherical sacs connected by medial ducts that led to the main pharynx, as well as by lateral ducts that opened to the exterior as pharyngeal pores. Interestingly, the caudal shift of the pores appeared to exceed that of the pharyngeal baskets themselves (Figure 4C). Although it was not determined in this study whether this topography points to any mechanism behind the caudal shift, it seems unlikely; at least the positions of the pores were not shifted simply as a secondary effect of being dragged by the caudal movement of the pharyngeal baskets.

It is important to note that the distribution of the coelom perfectly coincided with that of the stage 53 embryo described above, and thus the pericardium was also shifted caudally and was dorsally connected to the peritoneal cavity that was restricted caudal to the pharynx (Figure 4B, C). As a result, the elongated part of the pharynx appeared as a coelom-less domain, containing increasing numbers of suprapharyngeal somites, the morphological configuration of which somewhat resembled the neck of amniotes (Figures 5C, 6A-C).

The development of the vagus and glossopharyngeal nerves also followed the modification of the pharynx (Figure 3D–F). Until stage 50, the morphological patterns of these nerves in relation to the spinal nerves and the entire pharynx coincided almost perfectly with those in gnathostome embryos at comparable stages (Figure 3D, E). At stage 53, the roots of these cranial nerves overlapped the rostral spinal nerve ganglia (Figure 3F). The overall morphology of the peripheral nerves in the *E. burgeri* embryo resembled that of a *Myxine* embryo described by Holmgren [13]. It is conceivable, therefore, that not only did the posterior part of the pharynx move posteriorly, but also that the spinal nerves (and myotomes; Figures 2F and 3C) shifted their positions rostrally. In the present study, it could not be clarified how this rostral shift took place. The elongation of the vagus nerve around this stage in particular was substantial; it ran caudally along the dorsolateral aspect of the pharyngeal wall, from the hindbrain to the posterior pharynx, to innervate pharyngeal arches 4–11 (Figure 4B, C). In the adult, the main trunk of the vagus nerve was always found dorsal to the pharynx, and each branch passed ventrally in each arch to innervate the pharyngeal muscles (data from *E. burgeri*, not shown). No epibranchial placodal contribution was observed at this stage, but putative epibranchial placodes have been suggested in the pan-placodal domain of the earlier hagfish embryo [10]. Curiously, the above noted part of the vagus represents the proximal part of the nerve, which in gnathostomes and the lamprey lies more superficially (see [17]). Thus the glossopharyngeal and vagus nerves are located unusually medial with respect to the position of somatic components (see below).

**Figure 7:**
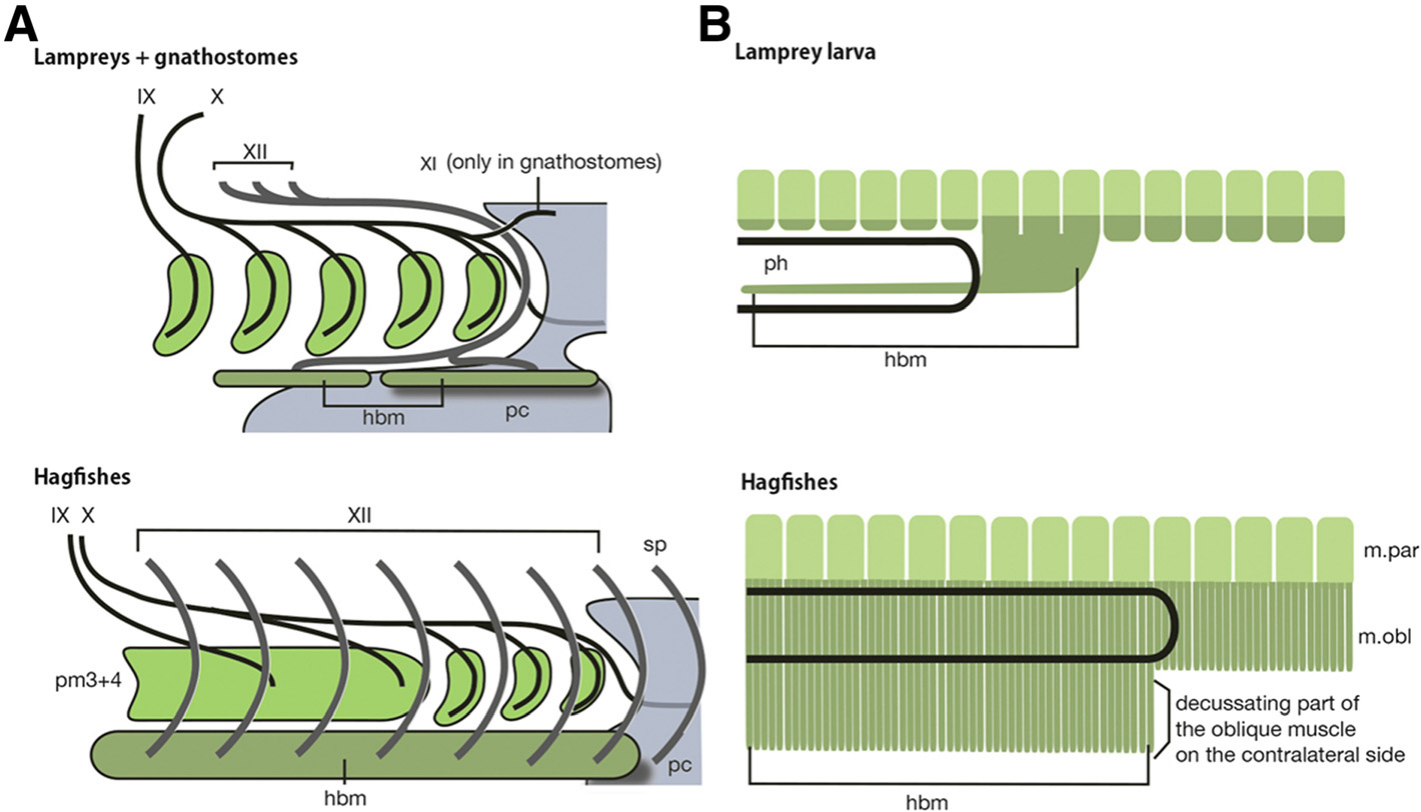
**Comparative morphology of the hagfish. A**: Differences in anatomical patterns between the hagfish and other vertebrates. Generally in vertebrates, hypoglossal or occipitospinal nerves (XII) pass along the posterior edge of the pharynx, and when the accessory nerve (XI) is present, nerve XII passes medial to nerve XI and lateral to the vagus nerve (X) to innervate the hypobranchial muscles (hbm). Thus nerve XII does not pass within the lateral body wall. In the hagfishes, putative hypobranchial muscle is assumed to arise in the rostral part of the ventral muscle that continues posteriorly into the rectus muscle in the trunk. Here, nerve XII does not form a single nerve trunk, but segmental occipitospinal nerves are shifted rostrally and no longer circumvent the pharynx caudally. However, this nerve still lies in the neck lateral to nerve X, as seen in the lampreys and gnathostomes. This peculiar morphology in the hagfish is thought to be due to a secondary modification of embryonic development, which is regarded as an autapomorphy for the hagfish. **B**: Homology of the hypobranchial muscle in the hagfish. The ventral somitic muscles of the hagfish can be seen as ventrally overgrown *Lbx1*-positive somitic muscles (dark green) in the larval lamprey. In the hagfish scheme, the pars decussata on the contralateral side is flipped back to the original (left) side of the body, and suspended ventrally. On the basis of this similarity, the homologue of the hypobranchial muscle is identified in the rostral oblique muscle with pars decussata in the hagfish.

The adult anatomical patterns of the peripheral nerves were apparent in the pre-hatching–stage *E. atami* embryo (Figure 3G, H). Surprisingly, the rostral spinal nerves were all located superficially with respect to the cranial nerve components (Figure 7A). Moreover, unlike in other vertebrate species, including the lamprey, the putative occipitospinal nerves in the hagfish never formed a single nerve trunk, as does a typical hypoglossal nerve, but remained as segmental spinal nerves (Figure 7A, B). This pattern is consistent with the even ventral growth of the ventral myoblasts in the hagfish, which do not show any “hypoglossal cord”–like structures (see below).

### Development of ventral somatic muscles

As described above, as far as the pharynx, coelom, and cranial nerves are concerned, the embryonic patterns are highly conserved among all vertebrates, and that pattern is also conserved in younger stages of the hagfish. However, the hagfish embryo stands out especially in terms of the developmental morphology and timing of the hypobranchial muscle and its innervating nerves—the hypoglossal, or occipitospinal nerve, as is described below.

Because the hypoglossal nerve of the typical morphological pattern is not observed in the hagfish, the identification of the hypobranchial muscle depends on its topographical and relative positions. Similar to the hypobranchial muscle in the lamprey, the hagfish has an anteroposteriorly elongated superficial muscle plate situated ventral and lateral to the tongue apparatus, with no overt segmental patterns (the obliquus and rectus muscles; [24]; Figure 1). It is however puzzling that these superficial muscles continued posteriorly along the entire length of the trunk. Therefore, there is apparently no clear gap between the hypobranchial muscle and abaxial trunk muscles in the hagfish (see below; [35,36]).

The primordium of the ventral muscles was first recognized in a stage 53 *E. burgeri* embryo, where it was located longitudinally along the ventral edges of somite-derived muscle plates, the primordium of parietal muscles (Figure 2F). In the histological sections, the parietal muscle anlage was seen as the direct growth of the myotome strongly expressing the *MyHCA* gene (Figures 5A, C, D and 6A–C), and the common anlage for the oblique and rectus muscles (ORM) was found laterally attached to the ventrolateral edge of the parietal muscle anlage (Figure 5A, C, D), prefiguring the relative positions of the two muscle groups in the adult (Figure 1). The somitic origin of the ORM was apparent, as the muscle anlagen were still found dorsal to the lateral body wall and lateral to the peritoneal cavity (Figure 5A, B). Unlike in the lamprey [37], these muscle primordia appeared to invade into the lateral body wall (Figure 5B), which was already filled with *HandA*-expressing mesenchyme (Figure 5B). We therefore provisionally regard the ORM as the “abaxial muscles” in the trunk of the hagfish. Although a ventrally projecting process was growing from the rostralmost part of the ORM anlage, it never formed an overt arch-like cell population of myoblasts resembling the hypoglossal cord that is seen in other vertebrate embryos. If this ventral process indicates the hypoglossal cord homologue, then the overall morphology of the hagfish embryo at this stage had already been modified in a hagfish-specific manner, implying that hypobranchial muscle development is relatively delayed in hagfish development.

At stage 60, differentiation of obliquus and rectus muscles was apparent (Figure 6D–F). The rectus muscle primordium was seen as an inner cell mass adhering to that of the obliquus, and consisted of bilateral paired strips of muscles along the longitudinal axis. However, at the level of the heart primordium that had not yet been incorporated within the body, these paired strips were widely separated from each other to allow the pericardium to protrude ventrally together with the heart (Figure 6D). Previous studies have shown that the mucous glands of the adult hagfish penetrate the oblique muscle (Figure 1). Here, the mucous gland first arose as a longitudinal cord of epithelium running ventral to the ORM anlage at stage 53 (Figure 6A–C). At this stage, there was no communication between the glands and the exterior. By stage 60, the glands had become separated into single epithelial spheres with ducts that were open to the body surface, thereby penetrating the oblique muscle, as seen in the adult (Figures 1C and 6D–F). It was therefore not determined in this study whether the opening of the ducts takes place before or after the ventral growth of the obliquus muscle anlage. A similar penetration was also found in the gill pores (Figure 6C). Because the gill pores had already become open to the exterior by stage 53, there would have been no process of penetration, but the ORM anlage grew ventrally, circumscribing the gill pores, between stages 53 and 60 (Figure 6).

## Discussion

Aside from the rostralmost part of the craniofacial region and the central nervous system, the vertebrate embryonic body consists of two major, conspicuously different domains: the pharyngeal arches and the trunk. The trunk is characterized by the presence of segmented paraxial mesodermal blocks, or somites, as well as the lateral plate–derived coelomic cavity. The outer wall of the cavity, which is called the lateral body wall, is derived from the somatopleure. The lateral body wall is occupied by hypaxial muscles that are primarily derived from somites. However, some somitic muscles are located neither in the paraxial domain nor in the lateral body wall; limb and hypobranchial muscles are in this category. Being derived from occipital or rostral myotomes, the hypobranchial muscle precursors migrate along the interface between the pharynx and body cavity (pericardium), together with the hypoglossal nerve anlage, to reach the oral floor in jawed vertebrates.

This embryonic pattern has been described for various vertebrate species [38-51]. In the lamprey, similar muscle precursors arise from rostral somites, once migrate caudally and ventrally along the caudal end of the pharynx in the rostralmost part of the body wall, to grow rostrally to reach the pharyngeal wall [25,28]. Thus, the myotomal muscle precursors nor the hypoglossal axons (somatic elements) do not typically enter into the pharyngeal arches [17,52]. The pharynx and trunk therefore not only stand out conspicuously in their morphological features, but also represent distinct developmental modules, with distinct developmental environments that favor specific sets of morphological elements. This is not unique; after all, the anatomical modules known as “visceral” or “somatic” have their embryonic backgrounds, as first recognized by van Wijhe [53].

The above distinction is clearly represented by the morphological patterns of a subset of cranial nerves distributed in the pharyngeal arches. These nerves, also known as “branchiomeric nerves” (cranial nerves V, VII, IX, and X) are primarily associated with pharyngeal arches, whereas spinal nerves belong to the trunk, exhibiting a metameric pattern in alignment with that of somites. The morphological pattern of the branchiomeric nerves is primarily characterized by their lateral position, determined by the position of the epibranchial placodes as well as the dorsolateral migratory pathway of the cephalic crest cells that prefigure the proximal nerve roots (reviewed by [17]). The dorsal root ganglion of the spinal nerve, on the other hand, is patterned more medially, medial to the dermomyotome. At the head-trunk interface, the mediolateral relationship between the vagus and hypoglossal nerves is reversed and the hypoglossal nerve comes towards the surface, whereas the pathway of the vagus switches from lateral to medial, growing caudally within the medial body wall along the esophagus [17,49]. This anatomical relationship is recapitulated in the lamprey, implying that this pattern is very ancestral, possibly dating back at least to the latest common ancestor of cyclostomes and gnathostomes [17].

In the hagfish, however, the above-mentioned anatomical pattern is greatly modified, i.e., the postotic pharynx is translocated extremely caudally, leaving a coelomless axis in front. In terms of the absence of the coelom, as well as the presence of a large number of suprapharyngeal myotomes, this elongated part of the hagfish resembles the “neck” of amniotes, probably as a homoplasy. The ventral portion of this “hagfish neck” is occupied by a cyclostome-specific structure, the lingual apparatus. This “cyclostome tongue” represents another homoplasy; unlike the somite-derived tongue in gnathostomes, it is a highly specialized organ derived from the mandibular arch [11,24,54]. The vagus nerve is extremely extended anteroposteriorly, together with the glossopharyngeal nerve at the level of pharyngeal arches 3 and 4, situated medial to the spinal nerve as well as trunk muscles. The results of the present study indicate that the initial topographical relationships among the pharynx, coeloms and cranial nerves (except the hypoglossal) are perfectly matched between the hagfish and the lamprey and gnathostomes.

Evolutionarily, the hagfish-specific peculiarity can be explained most parsimoniously as secondarily introduced changes unique in the lineage of hagfishes: since the morphological pattern of the lamprey hypobranchial/neck region resembles that of the gnathostomes, the evolutionary polarity suggests the apomorphic nature of the hagfish condition. Therefore, the ORM in the hagfish most likely represent secondary fusion or assimilation of hypobranchial muscles and abaxial muscles in the trunk, and not a primitive state before the separation of these two groups of muscles. This assumption simultaneously suggests that both the occipitospinal nerves and hypobranchial muscles had already been acquired before the split between cyclostomes and gnathostomes, more than 500 million years ago (reviewed by [55]). It should nonetheless be noted that the ancestral vertebrates may have possessed the hagfish ORM–like ventral muscles in the trunk that would not have been differentiated into hypobranchial and abaxial muscles. As mentioned by Nishi [23], the hypobranchial muscle (=rectus cervicus) and rectus abdominis were thought to represent serial homologues. Developmentally as well, these muscle primordia resemble each other, especially in terms of local mesenchyme-dependent patterning [35,36], even if there exists a conspicuous difference in the source of connective tissues [56,57].

The vertebrate hypobranchial muscles are conspicuous primarily in extant jawed vertebrates, and are generally regarded as a highly specialized category of trunk skeletal muscle. Anatomically, as seen in the tongue and infrahyoid muscle complex in mammals, the hypobranchial muscles do not reside in the lateral body wall, but are situated directly outside of the visceral structures and oral cavity. Developmentally, they arise from several rostral somites, including those often called the occipital somites, and the myoblasts migrate for a long distance along the posterior edge of the pharynx and root of the pericardium, to arrive at the oropharyngeal floor. Although this pathway is recognized in the embryonic context as the rostralmost part of the lateral body wall, this environment contains cephalic crest–derived ectomesenchyme, which will later contribute to the formation of the connective tissue of the hypobranchial muscles [17,50,56-59].

Differentiation and patterning of these muscles are highly dependent on *Pax3* expression, and hypobranchial myoblasts and other long-distance–migrating myoblasts of somitic muscles, including limb muscles, express *Lbx1*-homologues (marker of migrating muscle precursor cells; reviewed by [60-67]. Not much is known about the developmental regulation of hypobranchial myoblasts, but hepatocyte growth factor (HGF) is distributed in the embryonic environment corresponding to the above summarized pathway, and *c-Met*, the gene encoding the receptor for HGF, is expressed in the myoblasts, implying that HGF signaling may be involved in the pathway regulation [61]. Due to postembryonic changes, especially the retraction of the coelom and neck formation that proceed from anterior to posterior, mature hypobranchial muscle and hypoglossal nerve in late embryos of jawed vertebrates are no longer found in the body wall.

In the lamprey, although a typical hypobranchial muscle does not appear, its possible precursor, or homologue, has been identified and named the “hypobranchial muscle” for its position ventral to the gill pores. Similarly, the nerves that innervate the hypobranchial musculature have been termed the “occipitospinal nerves” or “hypoglossal nerve” [25,28,68-70]. This homology has long been assumed by comparative embryologists. Unlike in jawed vertebrates, the hypobranchial muscle of the lamprey is segmented along the anteroposterior axis, with each segment not directly aligned with a dorsal myotome, but rather spanning two successive branchial arch skeletons. Thus, this segmental configuration, unique to the lamprey, does not reflect its innate developmental pattern, but is very likely a derived feature, adapted for pure mechanical function.

Developmentally, the lamprey hypobranchial muscle appears to be derived from rostral myotomes, except, possibly, for the first two or three segments, which differentiate into supraoptic and infraoptic myotomes, which are other cyclostome-specific muscles (as for its potential homology with the cucullaris muscle, see [71]; unpublished data by [72]). The hypobranchial muscle in the lamprey develops rather late in embryogenesis, possibly as the direct elongation of the ventral edge of myotomes expressing *LjPax3/7* [73]. Although this anlage is a compact mass of cells, and does not appear to be composed of actively migrating mesenchymal myoblasts, its overall morphology is very reminiscent of the hypoglossal chord in other vertebrate embryos, even if it does not pass ventral to the pharynx and lateral to the pericardium. In lamprey and shark embryos, the hypobranchial muscle anlage is thought to grow as a direct extension of myotomes, rather than from migrating myoblasts [25,62,67,74], and the hagfish hypobranchial muscle seems to fall into this same category. Late expressions of *LjMyHC2*, *LjLbxA*, and *LjMRF-A* have also been detected in the hypobranchial muscle anlage in the lamprey [66,75]. Although most of these gene expression patterns are shared by the ventralmost part of the myotome in the lamprey, suggesting the possibility that ventral trunk muscle and hypobranchial muscles in the lamprey share common properties, there is also a distinction between these muscle anlagen both in terms of the morphology and growth rates [67,75]. In the hagfish, as far as we observed (mainly at histological levels), there was no clear distinction or difference found between the anterior and posterior parts of ORM anlagen.

Anatomically, however, there may at least be a clear distinction between the anterior and posterior part of the oblique muscles. Namely, the pars decussata only arises at the level of the pharynx and anterior (Figure 1C), and does not appear caudal to the pharynx. Thus, morphologically speaking, the hagfish oblique muscle is more ventrally extending in the rostral part, a configuration which is very reminiscent of the developing lamprey larvae [67] (Figure 7). It appears now very likely that the rostral part of the obliquus muscle with pars decussata on the contralateral side homologizes with the hypobranchial muscle in the lamprey (Figure 7, right). The anteroposterior distinction of the rectus muscle remains enigmatic. Only its association with the lingual apparatus is suggestive of its hypobranchial muscle–like nature. Further molecular and cellular level analyses would be necessary to identify the hypobranchial muscle homologues not only in the hagfish but also in the lamprey, where distribution of the cephalic neural crest–derived ectomesenchyme has not been fully understood.

As noted above, the rostral part of the ORM in the hagfish occupies a position equivalent to that of typical hypobranchial muscles in other vertebrates, but is not innervated by the typical occipitospinal nerve whose axons are found along the circumpharyngeal space (along the postpharyngeal arc). Instead, these muscles are innervated by the segmental spinal nerves that grow vertically from the rostral spinal cord. Strangely, this trajectory falls within the domain of third and fourth pharyngeal arches; these nerves violate the rule of mutual exclusion normally observed between the pharyngeal arches and trunk. It is true that for these nerves to reach the hypobranchial muscle, rather than growing caudally first for a long distance all the way to the posterior end of the branchial apparatus, to circumvent this apparatus by making an arch that grows ventrally, and then turns to take a rostralward pathway to come back to the muscle, taking a short cut would be much easier. There are several possible hypotheses to explain this exceptional morphology. First, there could still be unknown mesenchymal rearrangement that obliterates the typical head-trunk interface as seen in other vertebrates. Second, there is an unusual delay in the timetable for the development of the hypobranchial/hypoglossal system, and the difference in embryonic environments that establishes the head-trunk interface has long been deactivated by the time this organ is being patterned. Third, muscle precursor and nerve axons for the hypobranchial/hypoglossal system have acquired properties for patterning in the pharyngeal arch–like environment, which is unique to the hagfish.

Due to the limited amount of embryonic material, the lack of information about cyclostome myogenesis, and especially the inaccessibility to experimental embryology, we cannot easily evaluate the likelihood of the above hypotheses. It would, however, be worth comparing the anatomy and embryology of the amniote “neck”, which shows some similarity to the situation we have observed in the hagfish. In both mammals and birds, anteroposteriorly extended coelom-less domains are found between the skull and the shoulder girdle. These domains are characterized by the distribution of “neck muscles”, consisting of cucullaris and hypobranchial muscles, and by the distribution of the cephalic crest–derived ectomesenchyme, which provides connective tissues for the neck muscles [57].

Although the acquisition of the neck is not entirely comparable between birds and mammals (e.g., the entire set of postotic aortic arches as well as inferior ganglia, together with the parathyroid and thymus, shift caudally to the cardiac level in birds, whereas the dorsal part of the pharynx including the inferior ganglia remains close to the skull in mammals), some notable similarities exist, as seen, for example, in the superficial layer of the neck that is predominantly formed of second arch-derived cutaneous muscles (platysma muscle in mammals; m. constrictor superficialis coli, innervated by n. VII in avians). Thus, in amniotes, the hyoid arch becomes caudally expanded in the late pharyngular stage as a “collar”, to form the surface of the neck. By that time, the pharyngeal pores are mostly diminished, except for the ectodermal cervical sinus that continues production of nodose ganglionic neurons for the vagus nerve, while at the same time, pharyngeal arches 3 and posterior are being covered laterally by the second arch. The ventral surface of the neck, however, is not innervated by sensory fibers of the facial nerve, but in both mammals and birds, the cutaneous fibers distributed in that area originate from cervical spinal nerves (by way of ansa cervicalis in mammals). In sauropsids in particular, the cutaneous sensory fibers appear as segmentally arranged nerve nets, reflecting the presence of more clearly segmented cervical dermatomes in these animals when compared with those of mammals. This latter pattern of cervical nerve distribution is highly reminiscent of the spinal nerves’ innervation of the hypobranchial muscle in the hagfish (Figure 3H). These sensory branches penetrate the hyoid arch muscle, showing no sign of visceral/somatic distinction in their axonal morphology: they do not circumvent the hydoid arch–derived cutaneous muscles.

Similarly, the amniote hypoglossal nerves and muscles migrate in the lateral body wall (pericardium) only at their earliest phase of development; they are located close to the skull, far rostral to the caudal end of the neck, in the late embryonic to adult states. Thus, these nerves no longer indicate the caudal limit of the cephalic crest cells or the domain of head-like properties.

## Conclusions

We conclude that the hagfish hypobranchial/hypoglossal system could develop only by violating the basic embryonic architecture that governs the basic distribution of organs. For this to be possible, this group of cyclostomes has likely heterochronically shifted the timetable for hypobranchial/hypoglossal system development to the late phase of organogenesis, when spinal nerve axons are no longer blocked by pharyngeal arch derivatives, as the only method to maintain connections between nerves and muscles. Taking their feeding behavior and kinematics into consideration [76], it was probably necessary for hagfishes to have posteriorly open gill pores, considering their feeding behavior; specifically, it would have been necessary for them to keep their means of water and oxygen exchange clear while the head is lodged inside an animal cadaver during scavenging.

### Nomenclature

- *ao1-4*, aortic arches 1–4
- *e*, eye
- *gp1-6*, gill pores 1–6
- *h*, heart
- *I*, olfactory nerve
- *II*, optic nerve
- *IX*, glossopharyngeal nerve
- *IX + X*, glossopharyngeo-vagal nerve complex
- *lin*, lingual apparatus
- *lm*, lateral mesoderm
- *m.obl*, oblique muscles
- *mg*, mucous glands
- *m.par*, parietal muscles
- *m.rec*, rectus muscles
- *nc*, nasal cavity
- *nt*, notochord
- *oc*, oral cavity
- *ot*, otic vesicle
- *p1-4*, pharyngeal pouches 1–4
- *p.dec*, pars decussata of the oblique muscle originating from the contralateral side
- *pcd*, pharyngeocutaneous duct (only on the left side)
- *ph*, pharynx
- *pm1-5*, muscle precursors in pharyngeal arches 1–5
- *ps1-4*, pharyngeal slits 1–4
- *sc*, spinal cord
- *sk*, skin
- *sm*, somites
- *sp*, spinal nerves
- *ss*, spherical sac
- *V*, trigeminal nerve
- *VII*, facial nerve
- *V1*, ophthalmic nerve
- *V23*, maxillomandibular branches of the trigeminal nerve
- *X*, vagus nerve
